# Fungal Biofilms: Targets for the Development of Novel Strategies in Plant Disease Management

**DOI:** 10.3389/fmicb.2017.00654

**Published:** 2017-04-13

**Authors:** Federica Villa, Francesca Cappitelli, Paolo Cortesi, Andrea Kunova

**Affiliations:** Department of Food, Environmental and Nutritional Sciences, Università degli Studi di MilanoMilan, Italy

**Keywords:** fungal biofilm, non-fungicide management practices, biofilm resistance, bioactive natural compounds, non-biocidal antibiofilm compounds

## Abstract

The global food supply has been facing increasing challenges during the first decades of the 21^st^ century. Disease in plants is an important constraint to worldwide crop production, accounting for 20–40% of its annual harvest loss. Although the use of resistant varieties, good water management and agronomic practices are valid management tools in counteracting plant diseases, there are still many pathosystems where fungicides are widely used for disease management. However, restrictive regulations and increasing concern regarding the risk to human health and the environment, along with the incidence of fungicide resistance, have discouraged their use and have prompted for a search for new efficient, ecologically friendly and sustainable disease management strategies. The recent evidence of biofilm formation by fungal phytopathogens provides the scientific framework for designing and adapting methods and concepts developed by biofilm research that could be integrated in IPM practices. In this perspective paper, we provide evidence to support the view that the biofilm lifestyle plays a critical role in the pathogenesis of plant diseases. We describe the main factors limiting the durability of single-site fungicides, and we assemble the current knowledge on pesticide resistance in the specific context of the biofilm lifestyle. Finally, we illustrate the potential of antibiofilm compounds at sub-lethal concentrations for the development of an innovative, eco-sustainable strategy to counteract phytopathogenic fungi. Such fungicide-free solutions will be instrumental in reducing disease severity, and will permit more prudent use of fungicides decreasing thus the selection of resistant forms and safeguarding the environment.

## Introduction

Ensuring global food security is one of the greatest challenges facing humanity in the 21^st^ century. The intensification of world agriculture has to happen in an era, when climate is becoming less predictable, fossil fuel dependency needs to be cut, and cropland and water resources are shrinking or deteriorating (Alexandratos and Bruinsma, 2012; Popp et al., 2013). As a result, it is unclear how the growing demand for food can be achieved sustainably. Furthermore, the crops are constantly threatened by pests, pathogens and weeds. Indeed, several studies have estimated that, on average, 20–40% of the potential worldwide crop yield is lost each year due to pests and diseases (Oerke and Dehne, 2004; Savary et al., 2012; Popp et al., 2013). Hence, improved crop protection is one of the most important strategies to increase agricultural production and food availability.

Nowadays, management strategies integrate and coordinate a variety of approaches, from cultural practices, the use of resistant or tolerant crop varieties to physical, biological and chemical control methods. Worldwide legislation has now adopted the principles of integrated pest, disease and weed management (IPM), and is promoting methods alternative to pesticides, such as the globally accepted International Code of Conduct on the Distribution and Use of Pesticides (FAO, 2014), the European Union Directive 2009/128/EC or the US Food Quality Protection Act (FQPA). However, local governments still struggle to put the IPM principles into practice, these often being reduced to only chemical control and the implementation of simple warning models for pesticide application.

To ensure global food security, our society requires durable means of managing plant diseases that would be more sustainable, less fungicide-dependent, ecologically safe and socially acceptable. To this end, research on the ecology of phytopathogens needs to provide the basic knowledge to support the development of new control strategies that could be integrated in the IPM practices. A key to understanding the ecology of plant pathogens lies in determining their mode of growth and behavior, which provide microorganisms with survival advantages and increased virulence.

It is becoming increasingly evident that phytopathogens do not interact with the plant as individual entities, but rather at population level, in which microorganisms are social, and engage in complex behavior in response to the surface, other organisms and the extracellular environment. In other words, many plant pathogens form biofilm. The important hallmarks of a biofilm-based infection are increased resistance to conventional biocides, and their capacity for evading the host defenses (Ramage et al., 2012; Balcázar et al., 2015).

The formation of biofilms is not limited to the bacterial world, but rather includes fungal pathogens (Fanning and Mitchell, 2012; Borghi et al., 2015). The interest in fungal pathogenic biofilms relies mainly on the fact that some of the most devastating and universal crop diseases are caused by plant pathogenic fungi. Furthermore, although bacterial biofilms and their role in plant disease have been investigated in detail over a number of years (inter alia Ramey et al., 2004; Danhorn and Fuqua, 2007; Rudrappa et al., 2008; Bogino et al., 2013), much less is known about fungal biofilms. As a consequence, few options are available for controlling fungal pathogens, and chemical fungicides still dominate the market. However, chemical control is only one component of a multifaceted approach that should include more green strategies. Increasing reports of fungicide resistance in plant pathogens, restrictive regulations, and mounting concerns for human and environmental health issues resulting from excessive agrochemical use have stimulated the search for alternative, reliable disease management methods.

The recognition that many plant pathogens – including fungi – grow as biofilms, offers a possibility that they can be controlled adapting new methods and concepts developed by biofilm research.

In this perspective paper, we provide evidence to support the view that the biofilm lifestyle is critical for the pathogenesis of plant diseases, with an emphasis on fungal pathogens. We present an overview of the main factors limiting the durability of modern single-site fungicides, and we assemble the current knowledge on pesticide resistance, addressing this issue in the specific context of the biofilm lifestyle. We also examine the development and exploitation (or potential for exploitation) of a range of innovative, eco-sustainable strategies that take into account the new knowledge about biofilm ecology of pathogens and host-pathogen interactions. Such fungicide-free solutions will be instrumental in reducing disease severity, and will permit a more considerate use of single-site fungicides while decreasing the selection of resistant forms, safeguarding thus the environment.

## Current Understanding of Fungal Biofilms in Plant Diseases

Current developments in the ecology of plant–pathogen interactions reveal that surface-associated plant pathogens have morphological and physiological features consistent with a biofilm lifestyle. A biofilm is described as a microbial community attached to a surface and embedded in a self-produced matrix of polymeric substances.

**FIGURE 1:**
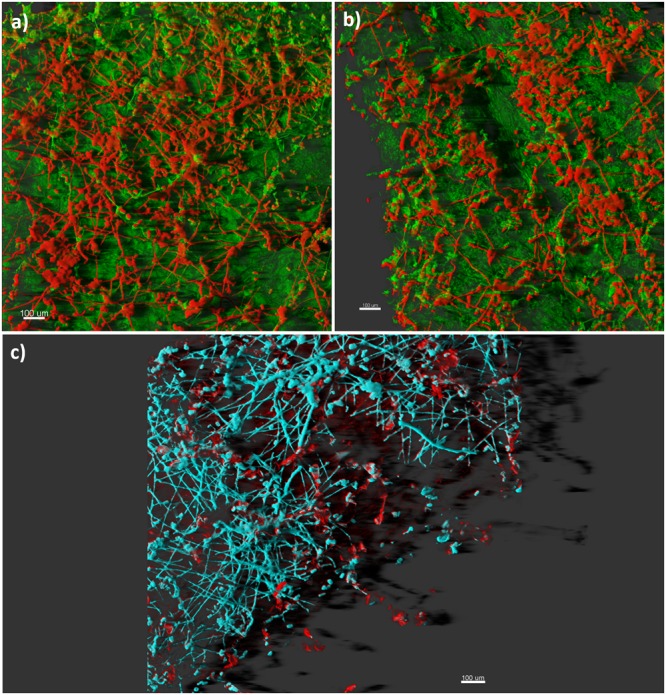
**Confocal laser scanning imaging of plant pathogenic fungi.** Panels **(a)** and **(b)** display a 3D projection of the fungal biofilm that has colonized leaf tissue: extensive hyphal networks and mycelial cords (red), and the plant tissue (green). Panel **(c)** shows a mature biofilm consisting of hyphal elements (cyano) encased in an extracellular polysaccharide matrix (red). Bars represent 100 μm.

Bacterial biofilms causing diseases in plants have been amply reported (*inter alia* Rojas et al., 2002; Newman et al., 2004; Quiñones et al., 2005; Danhorn and Fuqua, 2007; Chalupowicz et al., 2012). In contrast, plant pathogenic fungi have rarely been described to form biofilm, probably because filamentous fungi cannot fit precisely within the restrictive biofilm definition based on bacterial models. Nevertheless, according to a set of criteria reported by Harding et al. (2009), fungal growth associated with plant disease shows biofilm-like properties, such as extracellular polymeric materials (**Figure 1**), and population-level communication via diffusible extracellular signals. *Botrytis cinerea* growing on tomato stem was described as heavily layered, with extensive hyphal networks embedded in an exopolymeric matrix (Harding et al., 2010). Phenotypic changes in *Fusarium oxysporum* f. sp. *cucumerinum* growing on hard surfaces were reported, revealing a highly heterogeneous architecture composed of robust hyphae and extracellular polysaccharide materials (Peiqian et al., 2014). In this study, the cells in biofilm were less susceptible to heat, cold, UV light and three fungicides than their planktonic form. A number of diffusible extracellular signals, which are typical of a biofilm style, have been detected in fungi and oomycetes. Some of these signals modulate morphology (Hogan, 2006; Madhani, 2011; Barriuso, 2015). *Ophiostoma ulmi* (syn. *Ceratocystis ulmi*) produces molecules that repress fungal filamentation (Hornby et al., 2004). *Colletotrichum gloeosporioides* (syn. *Glomerella cingulata*) secretes a diffusible factor that suppresses mycelia formation (Lingappa and Lingappa, 1969; Bandara et al., 2012). *Ustilago maydis* secretes extracellular diffusible pheromones that induce a dimorphic switch from budding to a filamentous and infectious dikaryon form (Jones and Bennett, 2011). In addition, cyclic adenosine monophosphate (cAMP), a universal second messenger that regulates biofilm formation, is sufficient to modulate filamentation in many plant pathogenic fungi, including the rice blast pathogen *Pyricularia oryzae* (Ramanujam and Naqvi, 2010; McDonough and Rodriguez, 2011; Franck et al., 2013; Leng and Zhong, 2015; Marroquin-Guzman and Wilson, 2015). Finally, the oomycete *Phytophthora nicotianae* (syn. *P. parasitica*) produces a density-dependent signal that modulates the switch from planktonic form to biofilm, leading to massive zoospore encystment and cyst-orientated germination, and the production of extracellular matrix (Galiana et al., 2008; Kong et al., 2010; Theodorakopoulos et al., 2011).

## Traditional Biofilm Control Strategies: Problems of Fungicide-dependent Agriculture

The worldwide consumption of pesticides is currently about 1.5 million tons per year, and a total of 353 thousand tons of fungicides and bactericides were consumed per year on average across 77 countries (Liu et al., 2015). In 1999, fungicides in Europe accounted for 61% of the total pesticide consumption, mostly applied in viticulture and on cereal crops. Since then their use has decreased (Eurostat, 2007); new fungicides are applied at lower rates and over longer time intervals, their efficiency being much higher than that of the first preventative organic compounds. Currently, there is strong public awareness about the safety of chemical products used in plant protection. Indeed, the implementation of new regulations concerning the registration and sustainable use of plant protection products led, in the EU alone, to the removal of ca. 70% of the active ingredients used in agriculture before 1993 (European Commission, 2009). As a result, farmers now have to rely on a smaller number of relatively safer products. However, in some cases crop protection management strategies were left with just one – or only a few – active ingredients with different modes of action. This is particularly relevant for minor and specialty crops where only a limited number of fungicides have been registered (Rotteveel et al., 2011), and also for rice where, in the EU, the only products available to manage the blast pathogen *P. oryzae* are those containing azoxystrobin and tricyclazole, the latter currently excluded from the list of approved active ingredients and subjected to emergency approval (Kunova et al., 2013, 2014).

Nowadays, it has become increasingly more difficult to develop new fungicides. In 1995 the cost of discovery and the development of a new plant protection product was ca. $152 million, just 19 years later, in 2014, the cost had increased to $286 million, and to meet legislative requirements 3-times as many products had to be screened, increasing the average time of developing a new fungicide from 8.3 to 11.3 years (McDougall, 2016).

The evolution of fungicide resistance among fungal populations is another important factor driving the need to reduce our reliance on conventional fungicides. Most modern fungicides have a single-site mode of action, therefore the evolution of resistance in pathogen populations poses a major problem (Brent and Hollomon, 2007). Indeed, in many cases resistant pathogen populations emerged not long after the fungicides were introduced in practice in the field, and nowadays resistant pathogens are known to almost all the major groups of active ingredients (**Table 1**; Hayashi et al., 2001; Dubos et al., 2011, 2013; Fungicide Resistance Action Committee [FRAC], 2013, 2014; Lucas et al., 2015; Kunova et al., 2016). The best known is probably the case of strobilurin resistance (Quinone outside inhibitors, QoI; FRAC code 11), where resistant *Blumeria graminis* f. sp. *tritici* emerged only 2 years after the introduction of QoI-fungicides (Sierotzki et al., 2000). Moreover, multiple resistance has been observed for some fungal pathogens, in which they exhibit resistance to structurally and functionally unrelated compounds due to the overexpression of efflux pumps of the ATP-binding cassette (ABC) transporters (Leroux et al., 2013; Omrane et al., 2015). Efflux pumps became important in the 1990s as they were involved in the multidrug resistance of tumors, and later also in human pathogens, against clinical fungicides (Sanglard et al., 1995; De Waard et al., 2006; Ramage et al., 2012). To complicate this picture further, some authors have suggested that cross- and multidrug- resistance may be the driving force in the resistance development in fungi that are at the interface among agricultural, domestic, and hospital environments (De Lucca, 2007; Kaur et al., 2012). In fact, threats that fungi pose are not limited to plants; studies have shown that they are emerging as pathogens across diverse organisms, including soft corals (for example, sea-fan aspergillosis caused by *Aspergillus sydowii*), bees (the microsporidian fungus *Nosema* sp. associated with colony collapse disorder) and, last but not least, humans and animals (Sharon and Shlezinger, 2013; Soler-Hurtado et al., 2016). Today, *Aspergillus* and *Fusarium* conidia infect millions of susceptible individuals, causing allergies associated with asthma, allergic sinusitis and bronchoalveolitis, frequently with lethal consequences in immunocompromised patients (Żukiewicz-Sobczak, 2013). The plant pathogen *Cryptococcus neoformans* causes systemic human diseases contracted by inhalation of the infectious particle, which leads to primary pulmonary infections (Srikanta et al., 2014), while the corn smut fungus *U. maydis* causes skin lesions and peritonitis in both humans and animals (McNeil and Palazzi, 2012; Gauthier, 2015).

**Table 1:** Resistance of fungal plant pathogens to fungicides grouped by their mode of action (MoA).

Fungicide MoA	MoA subgroup	FRAC Code	No. resistant fungal pathogens (Fungicide Resistance Action Committee [FRAC], 2013, 2014)
A: NUCLEIC ACID SYNTHESIS	A1: RNA polymerase I: PA Fungicides (PhenylAmides)	4	36 (oomycetes)
	A2: Adenosine deaminase	8	2 (powdery mildews)
	**A3: DNA/RNA synthesis (proposed)**	**32**	**RESISTANCE NOT KNOWN**
	A4: DNA topoisomerase type II (gyrase): Carboxylic acids (Bactiricide)	31	1 (*Erwinia amylovora*)
B: MITOSIS AND CELL DIVISION	B1: β-tubulin assembly in mitosis: MBC Methyl Benzimidazole Carbamates	1	114
	B2: β-tubulin assembly in mitosis: N-phenylcarbamates	10	4
	**B3: β-tubulin assembly in mitosis: Benzamides**	**22**	**RESISTANCE NOT KNOWN**
	B4: Cell division (proposed)	20	1 (*Rhizoctonia solani*) – laboratory
	**B5: Delocalisation of spectrin like proteins**	**43**	**RESISTANCE NOT KNOWN**
C: RESPIRATION	**C1: Complex I, NADH oxidoreductase**	**39**	**RESISTANCE NOT KNOWN**
	C2: Complex II, succinate-dehydrogenase: SDHI fungicides	7	11
	C3: Complex III, cytochrome bc1: Quinone Outside Inhibitors	11	52
	C4: Complex III, cytochrome bc1: Quinone Inside Inhibitors	21	1 (*Phytophthora capsici*) – field
	C5: Uncouplers of oxidative phosphorylation	29	1 (*Botrytis cinerea*) – field
	C6: Inhibitors of oxidative phosphorylation. ATP synthase	30	1 (*Cercospora beticola*)
	C7: ATP production (proposed)	38	1 (*Gaeumannomyces graminis*) -field
	C8: Complex III, cytochrome bc1: Qx (unknown) site	45	NA
D: AMINO ACIDS AND PROTEIN SYNTHESIS	D1: Methionine biosynthesis (proposed; cgs gene): Anilinopyrimidines	9	3
	D2: Protein synthesis: Enopyranuronic acid antibiotic	23	2 – laboratory
	D3: Protein synthesis: Hexapyranosyl antibiotic	24	2
	D4: Protein synthesis: Glucopyranosyl antibiotic (Bactericide)	25	8
	D5: Protein synthesis: Tetracycline antibiotic (Bactericide)	41	3
E: SIGNAL TRANSDUCTION	E1: Signal transduction: Aza-naphthalenes	13	3 (powdery mildews)
	E2: MAP/Histidine-kinase in osmotic signal transduction (os-2, HOG1): Phenylpyrroles	12	6 – mostly laboratory
	E3: MAP/Histidine-kinase in osmotic signal transduction (os-1, Daf1): Dicarboximides	2	19
F: LIPIDS AND MEMBRANE SYNTHESIS	F2: Phospholipid biosynthesis, methyl transferase	6	2
	F3: Lipid peroxidation (proposed): Aromatic Hydrocarbons	14	4
	F4: Cell membrane permeability, fatty acids (proposed): Carbamates	28	8 (*Pythium* spp.)
	**F6: Microbial disrupters of pathogen cell membranes: Bacillus subtilis and the fungicidal lipopeptides produced**	**44**	**RESISTANCE NOT KNOWN**
	**F7: Membrane disruption (proposed): Plant extract**	**46**	**RESISTANCE NOT KNOWN**
G: STEROL BIOSYNTHESIS IN MEMBRANES	G1: C14 demethylase in sterol biosynthesis (erg11/cyp51): DMI fungicides	3	35
	G2: Δ14 reductase and Δ8 – Δ7isomerase in sterol-biosynthesis (erg24, erg2): Amines (‘morpholines’)	5	4
	G3: 3-keto reductase, C4-demethylation (erg27): Hydroxyanilides	17	1 (*Botrytis cinerea*) – field
	**G4: Squalene epoxidase in sterol biosynthesis (erg1): SBI class IV**	**18**	**RESISTANCE NOT KNOWN**
H: CELL WALL BIOSYNTHESIS	**H3: Trehalase and inositol biosynthesis: Glucopyranosyl antibiotic**	**26**	**RESISTANCE NOT KNOWN**
	H4: Chitin synthase: Polyoxins	19	6
	H5: Cellulose synthase: CAA fungicides. Carboxylic Acid Amides	40	6 (oomycetes)
I: MELANIN SYNTHESIS IN CELL WALL	I1: Reductase in melanin biosynthesis: MBI-R Melanin Biosynthesis Inhibitors – Reductase	16.1	1 (*Pyricularia oryzae*) – laboratory
	I2: Dehydratase in melanin biosynthesis: MBI-D Melanin Biosynthesis Inhibitors – Dehydratase	16.2	1 (*Pyricularia oryzae*) – field
P: HOST PLANT DEFENSE INDUCTION	**P1: Salicylic acid pathway: Benzothiadiazole BTH**	**P1**	**RESISTANCE NOT KNOWN**
	**P2: Benzisothiazole**	**P2**	**RESISTANCE NOT KNOWN**
	**P3: Thiadiazole-carboxamide**	**P3**	**RESISTANCE NOT KNOWN**
	**P4: Natural compound**	**P4**	**RESISTANCE NOT KNOWN**
	**P5: Plant extract**	**P5**	**RESISTANCE NOT KNOWN**
U: UNKNOWN MODE OF ACTION	Unknown: Cyanoacetamide-oxime	27	1 (*Plasmopara viticola*) – field
	Unknown: Phosphonates	33	4
	**Unknown: Phthalamic acids**	**34**	**RESISTANCE NOT KNOWN**
	**Unknown: Benzotriazines**	**35**	**RESISTANCE NOT KNOWN**
	**Unknown: Benzene-sulfonamides**	**36**	**RESISTANCE NOT KNOWN**
	**Unknown: Pyridazinones**	**37**	**RESISTANCE NOT KNOWN**
	**Unknown: Thiocarbamate**	**42**	**RESISTANCE NOT KNOWN**
	Unknown: Phenyl-acetamide	U6	1 (*Podosphaera fusca*) – glasshouse
	Actin disruption (proposed): Benzophenone	U8	1 (*Blumeria graminis* f.sp. *tritici*) – field
	Cell membrane disruption (proposed): Guanidines (dodine)	U12	1 (*Venturia inaequalis*)
	**Unknown: Thiazolidine**	**U13**	**RESISTANCE NOT KNOWN**
	**Unknown: Pyrimidinone-hydrazones**	**U14**	**RESISTANCE NOT KNOWN**
	**Oxysterol binding protein (OSBP) inhibition (proposed)**	**U15**	**RESISTANCE NOT KNOWN**
	**Complex III: cytochrome bc1, unknown binding site (proposed)**	**U16**	**RESISTANCE NOT KNOWN**
M: MULTI-SITE CONTACT ACTIVITY	Multi-site contact activity: Inorganic (copper)	M1	1 (*Xanthomonas axonopodis* pv. *citri*) – field
	**Multi-site contact activity: Inorganic (sulfur)**	**M2**	**RESISTANCE NOT KNOWN**
	Multi-site contact activity: Dithiocarbamates and relatives	M3	2 – laboratory
	Multi-site contact activity: Phthalimides	M4	1 (*Botrytis cinerea*) – laboratory, glasshouse
	Multi-site contact activity: Chloronitriles (phthalonitriles)	M5	1 (*Botrytis cinerea*) – laboratory
	Multi-site contact activity: Sulfamides	M6	1 (*Botrytis cinerea*) – laboratory
	Multi-site contact activity: Guanidines	M7	4
	**Multi-site contact activity: Triazines**	**M8**	**RESISTANCE NOT KNOWN**
	**Multi-site contact activity: Quinones**	**M9**	**RESISTANCE NOT KNOWN**
	**Multi-site contact activity: Quinoxalines**	**M10**	**RESISTANCE NOT KNOWN**
	**Multi-site contact activity: Maleimide**	**M11**	**RESISTANCE NOT KNOWN**

Moreover, the use of agricultural fungicides may result in the development of resistance in human pathogens, as suggested for *A. fumigatus* and *C. albicans* resistant to azoles, where the efflux pumps have been clearly involved in the biofilm resistance (Verweij et al., 2009; Snelders et al., 2012; Lelièvre et al., 2013; Bowyer and Denning, 2014; Faria-Ramos et al., 2014). Genes encoding drug efflux pumps have been reported to be differentially regulated in biofilms during the development and upon the exposure to antimicrobial agents, being predominantly expressed in the early phases and not in mature biofilms (Ramage et al., 2002; Mukherjee et al., 2003; Bueid et al., 2010).

However, efflux pumps are not exclusive determinants of fungal biofilm resistance. Biofilm, due to its cell density and matrix acting as a barrier, may impede the fungicide penetration. Although prevention of penetration is no longer believed to be a significant factor, binding fungicides to components of the biofilm matrix or to fungal membranes may also obstruct their penetration (Apoga et al., 2001; Doss et al., 2003). Positively charged pesticide molecules that bind to negatively charged biofilm matrix polymers might be delayed in their penetration through biofilm. High population densities and proximity of cells in biofilms also increases the chances for genetic exchange among microbial species converting biofilms in hot spots of biocide resistance (Fanning and Mitchell, 2012; Soanes and Richards, 2014; Balcázar et al., 2015).

Another efficient resistance strategy is the production of dormant structures within the biofilm matrix, such as spores, which help fungal plant pathogens to survive unfavorable conditions (Nadal et al., 2008; Gauthier, 2015). The reduced metabolic rate of these dormant structures makes them less sensitive to pesticides compared to active fungi, as widely described for bacterial biofilm (Jabra-Rizk et al., 2004; Van Acker et al., 2014).

These examples demonstrate that plant pathogen resistance, which could also result in treatment failure, could be associated with the ability of fungi to develop biofilm.

## New Ecologically Friendly Strategies to Manage Fungal Biofilms in Plant Diseases

In current agricultural production, high yields and healthy crops cannot be achieved without the chemical control of fungal diseases. Because of the many issues concerning fungicides, there is an urgent need for the development of new, efficient and environmentally safe strategies. The goal of these eco-friendly pesticide-free approaches is to prevent plant disease in the first place, not just to replace fungicides; this will help preserve the efficacy of fine fungicides currently on the market, and avoid as much as possible resistance development.

The concept of biofilm in plant pathogenic fungi offers the opportunity to exploit new environmentally friendly agricultural practices. It is reasonable to expect that interfering with the key-steps that orchestrate the genesis of virtually every biofilm (e.g., attachment, cell-to-cell communication, dispersion) could provide a way for new preventive strategies that do not necessarily exert lethal effects on cells, but rather sabotage the propensity for a biofilm lifestyle (Villa et al., 2013b). As these substances do not act by killing the cells, they should not impose a selective pressure that would cause the onset of resistance (Villa and Cappitelli, 2013).

Sub-lethal concentrations of zosteric acid (ZA), a secondary metabolite from the seagrass *Zostera marina*, reduce fungal adhesion and play a pivotal role in affecting fungal biofilm thickness and morphology (Stanley et al., 2002; Villa et al., 2010, 2011). The cells remain metabolically active but are unable to form filamentous structures. Moreover, ZA extends the performance of antimicrobial agents, shows cytocompatibility with soft and hard tissues, low bioaccumulation potential and absence of toxicity on *Daphnia magna* (Villa et al., 2011; Polo et al., 2014). ZA affects oxidative balance by interacting with the NADH: quinone reductase (WrbA), an enzyme belonging to a family of flavoprotein quinone reductases widely distributed in fungi (Villa et al., 2012; Cattò et al., 2015). The involvement of ZA in oxidative stress response is particularly promising as an alternative to conventional control strategies, as shown by the importance of ROS in fungal development and pathogenicity of several phytopathogenic fungi (Heller and Tudzynski, 2011; Mir et al., 2015).

Caripyrin, a pyridyloxirane recently isolated from submerged cultures of the basidiomycete *Gymnopus montagnei* (syn. *Caripia montagnei*), was found to inhibit conidial germination and appressorium formation in *P. oryzae* without being cytotoxic, antibacterial and nematicidal (Rieger et al., 2010). *Candida* biofilm formation was reduced by 63–98% when sub-MIC levels of *Boesenbergia pandurata* (finger root) oil were used (Taweechaisupapong et al., 2010). Purpurin, a natural red anthraquinone pigment commonly found in madder root, blocked *C. albicans* yeast-to-hypha transition when used at a sub-lethal concentration by down-regulating the expression of hypha-specific genes and the hyphal regulator RAS1 (Tsang et al., 2012). Pomegranate extract and its major component ellagic acid have anti-biofilm activity against *C. albicans* at sub-inhibitory concentrations (Bakkiyaraj et al., 2013). It also disrupted pre-formed biofilms and inhibited germ tube formation. Sub-lethal concentration of the bulb extract of *Muscari comosum* reduced the adhesion of *C. albicans* and induced the dispersion of biofilm cells in a dose-dependent manner (Villa et al., 2013a).

Although most of these studies have been conducted on the fungal model *C. albicans*, it is reasonable to expect that also fungal phytopathogens using dimorphism as a virulence strategy, such as *U. maydis, Mycosphaerella graminicola, Taphrina deformans*, or *O. ulmi* (Nadal et al., 2008), may be affected by these compounds, highlighting the potential effectiveness of biocide-free approaches.

The potential of antibiofilm compounds at sub-lethal concentrations has been proved against phytopathogenic bacteria, demonstrating – to some extent – the feasibility of the proposed antifungal strategy. A recent study showed that salicylic acid attenuates biofilm formation, swimming motility and acyl homoserine lactone production by different plant pathogens such as *Erwinia amylovora, Pseudomonas corrugata, P. syringae* pv *syringae, Xanthomonas campestris* pv *campestris*, and *Pectobacterium carotovorum* (Lagonenko et al., 2013). D-leucine and 3-indoloacetonitrile, which have been shown to inhibit biofilm formation and virulence in human bacterial pathogens, effectively prevented biofilm formation by the causal agent of citrus canker *X. citri* subsp. *citri.* The compounds were effective on different abiotic surfaces as well as on citrus leaves at sub-inhibitory concentrations, by repressing the expression of chemotaxis/motility-related genes in the phytopathogen (Li and Wang, 2014).

Understanding the consequences of biofilm manipulation could provide new antifungal targets as well as an important insight into the role of the biofilm mode of life in regulating stress or fungicide resistance.

## Conclusion

Fungal pathogens of cultivated crops remain a threat to food security also in the 21^st^ century, and the incorporation of new alternatives to conventional fungicides in disease management provides a sustainable approach for disease prevention and management. This is especially true in the light of the major challenges of efficiently increasing and protecting crop yield, while maintaining economic profits and at the same time preserving human health and the environment.

Some examples presented in this perspective paper provide possible solutions to reduce reliance on conventional fungicides for crop protection. Antibiofilm compounds at sub-lethal concentrations have partially been explored in the past and in other scientific fields. However, the challenge now is to take this knowledge and apply it in the agricultural context to develop novel tools for managing disease and to understand which of these strategies have the best potential in various environmental scenarios. This constitutes a long-term challenge that requires interdisciplinary research to address major issues of relevance to both science and society.

## Author Contributions

FV and AK wrote the manuscript. FV performed the confocal microscopy. FC and PC participated in discussions and improved the manuscript. AK coordinated the collaboration of the authors. All authors read and approved the final manuscript.

## Conflict of Interest Statement

The authors declare that the research was conducted in the absence of any commercial or financial relationships that could be construed as a potential conflict of interest.
